# Masked Face Emotion Recognition Based on Facial Landmarks and Deep Learning Approaches for Visually Impaired People

**DOI:** 10.3390/s23031080

**Published:** 2023-01-17

**Authors:** Mukhriddin Mukhiddinov, Oybek Djuraev, Farkhod Akhmedov, Abdinabi Mukhamadiyev, Jinsoo Cho

**Affiliations:** 1 Department of Computer Engineering, Gachon University, Seongnam 13120, Republic of Korea; 2 Department of Hardware and Software of Control Systems in Telecommunication, Tashkent University of Information Technologies Named after Muhammad al-Khwarizmi, Tashkent 100084, Uzbekistan

**Keywords:** emotion recognition, facial landmarks, computer vision, deep learning, convolutional neural network, facial expression recognition, visually impaired people

## Abstract

Current artificial intelligence systems for determining a person’s emotions rely heavily on lip and mouth movement and other facial features such as eyebrows, eyes, and the forehead. Furthermore, low-light images are typically classified incorrectly because of the dark region around the eyes and eyebrows. In this work, we propose a facial emotion recognition method for masked facial images using low-light image enhancement and feature analysis of the upper features of the face with a convolutional neural network. The proposed approach employs the AffectNet image dataset, which includes eight types of facial expressions and 420,299 images. Initially, the facial input image’s lower parts are covered behind a synthetic mask. Boundary and regional representation methods are used to indicate the head and upper features of the face. Secondly, we effectively adopt a facial landmark detection method-based feature extraction strategy using the partially covered masked face’s features. Finally, the features, the coordinates of the landmarks that have been identified, and the histograms of the oriented gradients are then incorporated into the classification procedure using a convolutional neural network. An experimental evaluation shows that the proposed method surpasses others by achieving an accuracy of 69.3% on the AffectNet dataset.

## 1. Introduction

Understanding and responding to others’ emotions is crucial to interpreting nonverbal cues and the ability to read another person’s emotions, thoughts, and intentions. Humans use a variety of cues including voice intonation, word choice, and facial expression to interpret emotional states. Non-verbal cues, such as facial expressions, are essential in communication, but people who are blind or visually impaired are unable to perceive these cues [1]. Accurate emotion recognition is particularly important in social interactions because of its function in helping people communicate more effectively. For instance, how people react to their interactions with individuals is affected by the emotions they are experiencing. The inferential processes that are triggered by an emotional expression might then inform of subsequent thoughts and behaviors, as proposed by the emotion as social information paradigm [2]. If the observer notices that the person being observed is depressed because they cannot open the door, they may offer to assist. Recognizing people’s emotions correctly is critical because each emotion demonstrates unique information and feelings. If people in a meeting cannot recognize each other’s emotions, they may respond counterproductively to the attention.

Emotional mimicry, or the act of mirroring the nonverbal behaviors underlying an individual’s emotional expressions [3,4], has been shown to boost a person’s likeability and, consequently, the likelihood that they will like and be willing to form a relationship with that person. In light of this, it is essential to correctly label an interaction partner’s emotional expression, especially in an initial meeting. Individuals use different methods to convey their emotions, including their faces [5,6]. Different emotions call for different sets of facial muscles [7]; therefore, the face serves as a particularly rich source of affective data. Thus, it is up to observers to translate information shared by facial clues in order to visualize the emotional experiences of others. Our eyebrows, eyes, nose, and mouth are all highly informative. Covering the mouth and a portion of the nose, as is standard with face masks, reduces the variety in available facial cues. As a result, they may impair the observer’s ability to accurately identify the emotions conveyed by a person’s facial expressions [8]. A survey has shown that adults’ identification accuracy dropped when asked to identify faces with their mouths covered [9].

The demand for intelligent technologies to decide a potential client’s desires and needs and select the best action approach has rocketed with the widespread adoption of intelligent technologies in modern life and with the growth in this industry. Furthermore, computer vision and deep learning approaches are implemented in almost every engineering area and social sphere, such as emotion recognition [10,11], manufacturing [12,13], text and speech recognition [14,15], and medical imaging [16,17]. In spite of the significant success of conventional facial emotion recognition approaches based on the extraction of handmade characteristics, during the previous decade, researchers have turned their focus to the deep learning approach due to its excellent automatic recognition power. Researchers have recently published review articles examining facial emotion recognition approaches. Mellouk et al. [18] presented an overview of recent improvements in facial emotion detection by recognizing facial expressions using various deep-learning network structures. They summarize findings from 2016 to 2019, together with an analysis of the issues and contributions. This review’s authors found that all of the papers they examined included some form of image preprocessing, which included methods such as cropping and resizing images to shorten the training period. In addition, to improve the image variety and solve the over-fitting issue, data augmentation and normalization of spatial and intensity pixels were applied. Saxena et al. [19] collected data, analyzed the essential emotion detection algorithms created over the past decade, and settled on the most effective strategies for identifying emotions conveyed in facial expressions, written material, physiological signals, and spoken words. More than a hundred sources, such as surveys, studies, and scholarly articles, were used to understand the work. Features, datasets, and methods utilized for emotion recognition were analyzed and compared. Chul Ko [20] also discussed a cutting-edge hybrid deep-learning strategy, which uses a convolutional neural network (CNN) to analyze the spatial properties of a single frame and a long short-term memory (LSTM) to explore the temporal features of multiple frames. As the report draws to a close, it provides a brief overview of publicly available evaluation criteria. It describes how they stack against benchmark results, which serve as a standard for quantitative comparisons among facial emotion recognition studies. In 2020, following a review of over 160 academic works, Dredzickis et al. [21] categorized emotion identification techniques by summarizing traditional approaches to the problem and various courses taken to increase confidence in the results. This work also presents an engineering perspective on the accuracy, sensitivity, and stability of emotion identification techniques.

In this study, we proposed a facial emotion recognition method from masked face images using computer vision and deep learning approaches for blind and visually impaired (BVI) people. It was developed because BVI people enjoy being socially active in communicating with others. The suggested approach is able to recognize facial emotions in dark conditions and contains two domains: (1) reducing noise and image improvement constraints via a low-light image increase utilizing a two-branch exposure-fusion design based on a CNN [22,23]; (2) emotion recognition based on facial landmarks and CNN. Our proposed approach starts with developing a deep learning model for recognizing facial expressions based on the AffectNet [24] dataset. Initially, the model was trained on the original facial emotion dataset. After that, the lower part of the face in the images was masked using the MaskTheFace algorithm [25] for re-training and transfer learning with the masked facial images. Our study’s next step involved using transfer learning to train a model for facial emotion recognition in covered faces with masks. Following developing a facial expression detection model, we utilized the MediaPipe face mesh technique to generate facial landmarks.

The contributions of this work are outlined as follows:

- The facial emotion recognition part of the smart glasses design was implemented to assist BVI people in understanding and communicating with people. Current smart glasses designs do not have the facial emotion recognition method in a low-light noise environment. It uses real-time audio results to inform users about their direct surroundings [22];
- We used a low-light image enhancement technique to solve the problem of misclassification in scenarios where the upper parts of the face are too dark or when the contrast is low;
- To recognize facial emotion, specific facial landmark modalities employ the MediaPipe face mesh method [26]. The results indicate a dual role in facial emotion identification. Specifically, the model can identify emotional states in either masked or unmasked faces;
- We created a CNN model with feature extraction, fully connected, SoftMax classification layers. The Mish activation function was adopted in each convolution layer. The use of Mish is a significant development that has the potential to enhance categorization precision.

This paper’s remaining sections are structured as follows. We review existing facial emotion recognition methods in Section 2. Section 3 outlines the data collection and modification and describes the proposed masked facial emotion recognition approach. The experimental results and analysis are presented and discussed in Section 4. In Section 5, we discuss the limitations and outcomes of our study and suggest future directions.

## 2. Related Works

### 2.1. Upper and Lower Parts of The Face

Based on previous research showing that facial expression recognition is decreased when a portion of the face is unobservable [27], it stands to reason that emotion recognition is affected by masking the face with various face coverings. Various current works [28,29,30,31,32] have shown this effect. Although visual manipulation makes it possible to standardize emotional indication between the mask and no-mask scenarios [30], it can introduce input artefacts that could interfere with emotion recognition. In a big smile, for instance, the top portion of a mask may rise and the widening of the lips in surprise may expand it vertically; these changes in the lower half of the face’s features can be seen as aspects that help convey mood. Furthermore, a person’s facial expression of emotion may shift when they cover their face. Photo editing to artificially set face covering can skew results and prevent a naturalistic study of the effects of masks on facial expression identification.

**Figure 1:**
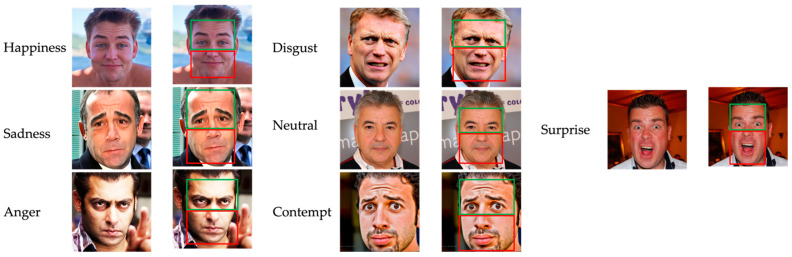
Example of seven facial emotions for upper and lower parts of the face: green rectangle box for upper part; red rectangle box for lower part.

Recent studies that have examined masks and facial emotion recognition have found that wearing a mask reduces the accuracy of emotion recognition. However, this decrease in accuracy is not uniform across all facial expressions. For instance, facial emotion recognition insufficiencies for Happiness, Sadness, Disgust, and Anger were found, but not for Fear or Neutral emotions [29,30]. First, covering the lower features of the face, such as the mouth, cheeks, and nose, with masks has different effects on different facial expressions, as experimented with using “bubbles” in the study [33]. In addition, other approaches imply that the primary informative sections of the face vary between facial expressions [34,35]. In contrast, analyses of masking the face have shown differences throughout expressions in the outcomes of hiding the eye against mouth parts [36,37]. Figure 1 shows example images of seven facial emotions.

Studies based on bubbles have shown that the lower parts of the face provide the most details about a person’s emotional condition when they are happy, surprised, or disgusted. The upper parts of the face provide the most details concerning a person’s emotional condition when they are afraid or angry, and the lower and upper parts provide the same information when the person is sad or neutral [34,35]. The best uniform effect from comparing the coverage of the lower and upper regions of the face is that covering the lower part disrupts recognition of happiness more compared to covering the upper part. At the same time, other emotions have varying results: for instance, the authors of [38] observed that covering the mouth interrupted emotions of disgust and anger more than eye covering; however, the author of [37] found the reverse trend.

### 2.2. Facial Landmarks

**Figure 2:**
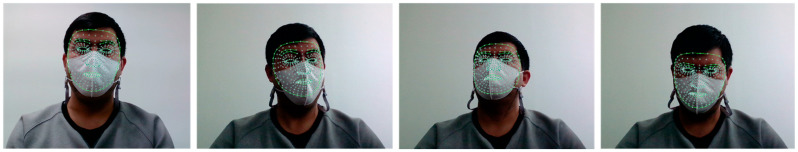
Example of facial landmark images.

Facial landmarks present valuable data for exploring facial expressions as shown in Figure 2. Yan et al. [39] proposed facial landmarks as action unit derivatives to describe face muscle motions. Other studies [40,41] have introduced a variety of landmark and image fusion processes. Hasani et al. [42] proposed a merging of videos and landmarks. The deformable synthesis model (DSM) was proposed by Fabiano et al. [43]. These algorithms demonstrate the effectiveness of the landmark feature; nonetheless, emotion identification algorithms employing landmark characteristics have been investigated rarely in recent years. This is not because the information offered by landmark features is insufficient, but rather because suitable techniques for extracting information from landmark features have yet to be chosen. Recently, Ngos et al. [44] introduced a graph convolutional neural network utilizing facial landmark features to identify points, and the edges of the graph were constructed by employing the Delaunay technique. Khoeun et al. [45] proposed a feature vector approach for recognizing emotions of masked faces with three key components. The authors used facial landmark identification to retrieve the characteristics of covered faces with masks, and upper facial landmark coordinates were used to identify facial expressions. Nair and Cavallaro [46] suggested a robust framework for detecting and segmenting facial landmark position to match face meshes to facial standards. First, face regions were segmented and landmark position was performed. Additionally, Hemang et al. [47] compared the 3D data of facial feature coordinates to the 2D coordinates acquired from a photo or live stream using Levenberg–Marquardt optimization and a projection matrix. By employing this strategy, the authors could identify the ideal landmarks and calculate the Euler angles of the face.

Various methods describe emotions based on a mixture of certain facial features, including the upper and lower features of the face. Existing methods that depend solely on action units are restricted by the need for more information from the bottom features of the face, resulting in a reduction in accuracy. Table 1 provides a comparison of the available techniques.

Current facial emotion recognition algorithms explained in [53,54,55] that rely on standard 68-landmark detection involve searching the whole picture to locate the facial contours and then labeling the face with the positions of the 68 landmarks. The links between these landmarks are then analyzed. However, these approaches rely heavily on the interaction between the bottom and top features of the face; hence, accomplishment is interrupted when the bottom features of the face are invisible, resulting in roughly 40% of the information being unavailable. For these face-based algorithms, every pixel in the identified faces is utilized to learn and categorize emotions, resulting in a significant degree of computing complexity and time. Face-based approaches have the disadvantage of utilizing all pixels, which are irrelevant to the operation. Furthermore, these unnecessary pixels interrupt the process of training, resulting in low accuracy and great complexity.

## 3. Materials and Methods

### 3.1. Datasets for Facial Emotion Recognition

The original MultiPie [57], Lucey et al. [58], Lyons et al. [59], and Pantic et al. [60] datasets of facial expressions were recorded in a laboratory setting, with the individuals acting out a variety of facial expressions. Using this method, we created a spotless, high-quality repository of staged facial expressions. Faces in pictures may look different from their unposed (or “spontaneous”) counterparts. Therefore, recording emotions as they happen became popular among researchers in affective computing. Situations such as this include experiments in which participants’ facial reactions to stimuli are recorded [60,61,62] or emotion-inducing activities are conducted in a laboratory [63]. These datasets often record a sequence of frames that researchers may use to study expressions’ temporal and dynamic elements, including capturing multi-modal impacts such as speech, bodily signals, and others. However, the number of individuals, the range of head poses, and the settings in which these datasets were collected all contribute to a lack of variety.

Therefore, it is necessary to create methods based on natural, unstaged presentations of emotion. In order to meet this need, researchers have increasingly focused on real-world datasets. Table 1 provides a summary of the evaluated databases’ features across all three affect models: facial action, dimensional model, and category model. In 2017, Mollahosseini et al. [24] created a facial emotion dataset named AffectNet to develop an emotion recognition system. This dataset is one of the largest facial emotion datasets of the categorical and dimensional models of affect in the real world. After searching three of the most popular search engines with 1250 emotion-related keywords in six languages, AffectNet gathered over a million photos of people’s faces online. The existence of seven distinct facial expressions and the strength of valence and arousal were manually annotated in roughly half of the obtained photos. AffectNet is unrivalled as the biggest dataset of natural facial expressions, valence, and arousal for studies on automated facial expression identification. The pictures have an average 512 by 512 pixel resolution. The pictures in the collection vary significantly in appearance; there are both full color and gray-scale pictures, and they range in contrast, brightness, and background variety. Furthermore, the people in the frame are mostly frontally portrayed, although items such as sunglasses, hats, hair, and hands may obscure the face. As a result, the dataset adequately describes multiple scenarios as it covers a wide variety of real-world situations.

In the ICML 2013 Challenges in Representation Learning [64], the Facial Expression Recognition 2013 (FER-2013) [65] database was first introduced. The database was built by matching a collection of 184 emotion-related keywords to images using the Google Image Search API, which allowed capturing the six fundamental and neutral expressions. Photos were downscaled to 48 × 48 pixels and converted to grayscale. The final collection includes 35,887 photos, most of which were taken in natural real-world scenarios. Our previous work [56] used the FER-2013 dataset because it is one of the largest publicly accessible facial expression datasets for real-world situations. However, only 547 of the photos in FER-2013 depict emotions such as distaste, and most facial landmark detectors are unable to extract landmarks at this resolution and quality due to the lack of face registration. Additionally, FER-2013 only provides the category model of emotion.

Mehendale [66] proposed a CNN-based facial emotion recognition and changed the original dataset by recategorizing the images into the following five categories: Anger-Disgust, Fear-Surprise, Happiness, Sadness, and Neutral; the Contempt category was removed. The similarities between the Anger-Disgust and Fear-Surprise facial expressions in the top part of the face provide sufficient evidence to support the new categorization. For example, when someone feels angry or disgusted, their eyebrows will naturally lower, whereas when they are scared or surprised, their eyebrows will raise in unison. The deletion of the contempt category may be rationalized because (1) it is not a central emotion in communication and (2) the expressiveness associated with contempt is localized in the mouth area and is thus undetectable if the individual is wearing a face mask. The dataset is somewhat balanced as a result of this merging process.

In this study, we used the AffectNet [24] dataset to train an emotional recognition model. Since the intended aim of this study is to determine a person’s emotional state even when a mask covers their face, the second stage was to build an appropriate dataset in which a synthetic mask was attached to each individual’s face. To do this, the MaskTheFace algorithm was used. In a nutshell, this method determines the angle of the face and then installs a mask selected from a database of masks. The mask’s orientation is then fine-tuned by extracting six characteristics from the face [67]. The characteristics and features of existing facial emotion recognition datasets are demonstrated in Table 2.

### 3.2. Proposed Method for Facial Emotion Recognition

**Figure 3:**
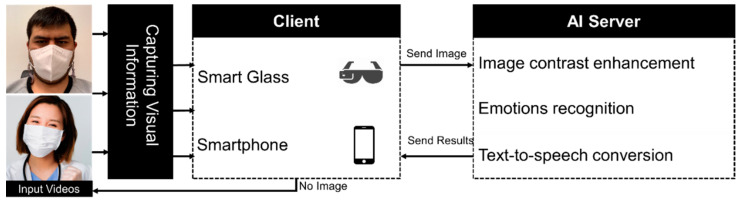
The general structure of the client-server architecture.

Our aim is to enhance the quality of life for the BVI people by making it easier for them to communicate socially with other human beings, both during the day and at night. Wearable smart glasses and a multipurpose system able to record pictures through a tiny camera and provide facial emotion recognition results using audio data to BVI people are the most practical means of achieving this aim. The system needs a solid CPU to quickly run deep CNN models for real-time emotion recognition. As a result, we proposed a client-server architecture wherein smart glasses and a smartphone are client devices while an AI server processes input video frames. The work proposed here presents a comprehensive, two-part deep learning framework for use in all stages of the learning process. The general design of the proposed system is shown in Figure 3. The local component uses Bluetooth to transmit data between a smartphone and the smart glasses. Meanwhile, the recorded images are transferred to the AI server, which processes them and then plays them back to the user as an audio file. Keep in mind that the hardware for smart glasses has both a built-in speaker for direct output and an earphone connector for the audio connection, allowing users to hear the responses of voice feedback sent from their smartphones.

The client-side workflow entails the following steps: first, the user establishes a Bluetooth connection between their smart glasses and a smartphone. Once this is done, the user may instruct the smart glasses to take pictures, and the smartphone will then receive those pictures. This situation better serves smart glasses’ power needs than a continuous video recording. The AI server then provides spoken input through headphones, a speaker system, or a mobile device. Despite the recent introduction of light deep CNN models, we still conducted face expression recognition tasks on an AI server rather than a wearable assistive device or smartphone CPU. The fact that smart glasses and smartphones are solely used for taking pictures also helps them last longer on a single charge.

The AI server is comprised of three primary models: (1) an image enhancement model for low-contrast and low-light conditions; (2) a facial emotion recognition model; and (3) a text-to-speech model for converting text results to audio results. In addition, the AI server component has two modes of operation, day and night, which are activated at different times of the day and night, respectively. The low-light picture-enhancing model does not operate in daylight mode. The following is how the nighttime mode operates: After receiving a picture from a smartphone, the system initially processes it with a low-light image improvement model to improve the image’s dark-area quality and eliminate background noise. After the picture quality has been enhanced, facial emotion recognition models are applied to recognize masked and unmasked faces, and a text-to-speech model is performed. The AI server sends back the audio results in response to the client’s request.

#### 3.2.1. Low-Contrast Image Enhancement Model

Pictures captured in low contrast are characterized by large areas of darkness, blurred details, and surprising noise levels compared to similarly composed images captured in standard lighting. This can happen if the cameras are not calibrated properly or if there is very little light in the scene, as in the nighttime or a low-contrast environment. Thus, the quality of such pictures is poor because of the insufficient processing of information required to develop sophisticated applications such as facial emotion detection and recognition. As a result, this subfield of computer vision is one of the most beneficial in the field and has drawn the interest of many scientists because of its significance in both basic and advanced uses, such as assistive technologies, autonomous vehicles, visual surveillance, and night vision.

**Figure 4:**
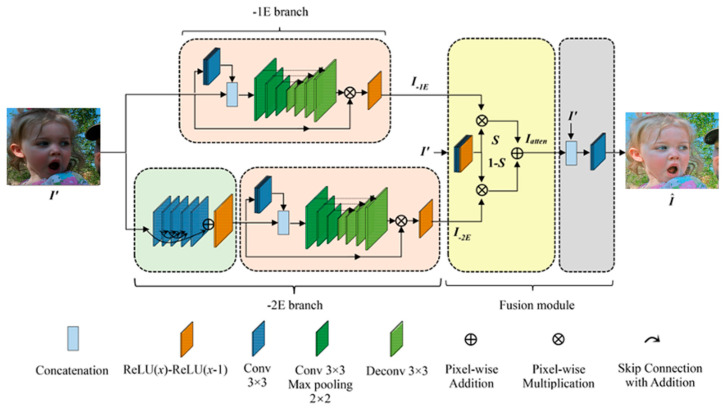
Structure of the network used in a model that improves images with low contrast [23].

An ideal and successful approach would be to deploy a low-light image enhancement model to allow the facial emotion recognition model to autonomously work in a dark and low-contrast environment. Recently, a deep learning-based model for improving low-light images has shown impressive accuracy while successfully eliminating a wide range of noises. For this reason, we implemented a low-light image improvement model using a CNN-based two-branch exposure-fusion network [26]. In the first stage of the low-light improvement process, a two-branch illumination enhancement framework is implemented, with two different enhancing methodologies used separately to increase the potential. An information-driven preprocessing module was included to lessen the deterioration in extremely low-light backgrounds. In the second stage, these two augmentation modules were sent into the fusion part, which was trained to combine them using a useful attention strategy and a refining technique. Figure 4 depicts the network topology of a two-node exposure-fusion system [23]. Lu and Zhang evaluated the two branches as −1E and −2E because the top branch is more beneficial for images in the measurement approach with only a level of exposure of −1E, while the second branch is more effective for images with an exposure level of −2E.

$F_{en}^{branch}$ independently creates the −1E branch and the main structure of the −2E branch without requiring an additional denoising procedure. The improvement module’s output is depicted as follows:

(1) $$I_{out}^{branch}=I_{in}^{branch}\circ F_{en}^{branch}\left(I_{in}^{branch}\right)$$

where branch ∈ {−1E, −2E}. The input and output pictures are denoted by ***I****_in_* and ***I****_out_*. Initially, four convolutional layers are applied to the input picture to extract its additional features, which are then concatenated with the input low-light images before being fed into this improvement module [23].

The −2E training branch is used to teach this component to identify the degree of picture degradation caused by factors such as natural noise in the preprocessing module. In order to explain the preprocessing module’s functionality, multilayer element-wise summations were used. The feature maps from the fifth convolutional layer, which used a filter size of 3 by 3, were added to the feature maps from the preceding layers to facilitate training. In addition, no activation function was utilized after the convolution layer; the input characteristics were scaled down to [0,1] using the modified ReLU function in the last layer. To be able to recreate the intricate patterns even in a dark environment, the predicted noise range was adjusted to (−∞, +∞).

*Out*(*x*) = *ReLU*(*x*) − *ReLU*(*x* − *1*) (2)

In the fusion module, the two-branch network improved results were integrated with the attention unit and then refined in a separate unit. For the −1E improved picture, the attention unit used four convolutional layers to produce the attention map ***S*** *= F_atten_*(***I′***), whereas the −2E image received the corresponding element 1 − *S*, with ***S***(*x*, *y*) ∈ [0,1]. By adjusting the weighted template, this approach seeks to continually aid in the development of a self-adaptive fusion technique. With the help of the focus map, we can see that the R, G, and B channels are all given the same amount of consideration. The following is the computed outcome from the ***I****_atten_* attention unit:

***I**_atten_* = ***I***_−1E_ ◦ ***S*** + ***I***_−2E_ ◦ (1 − ***S***) (3)

While this straightforward method produces improved pictures from both the −1E and −2E branches, there is a risk that some crucial details may be lost during the fusion process. Furthermore, noise levels may rise if a direct metric is employed. In order to fix this, ***I****_atten_* was combined with its low-light input and delivered to the ***F****_ref_* refining unit. The final improved picture formulization is as follows:

**Î** = ***F**_ref_* (*concat{**I**_atten_,**I′**}*) (4)

In this training, smooth, VGG, and SSIM loss functions were used. It is possible to utilize total variation loss to characterize the smoothness of the predicted transfer function in addition to its structural properties when working with a smooth loss. Smooth loss is calculated using Equation (5) and the per-pixel horizontal and vertical dissimilarity is denoted by $\nabla_{x,y}$.

(5) $$L_{SMOOTH}=\underset{branch\left\{-1E,-2E\right\}}{\sum }\|\nabla_{x,y}F_{en}^{branch}\left(I_{in}\right)\|$$

VGG loss was employed to solve two distinct issues. First, according to [23], when two pixels are bound with pixel-level distance, one pixel may take the value of any pixels within the error radius. This tolerance for probable changes in hues and color depth is what makes this limitation so valuable. Second, pixel-level loss functions do not accurately represent the intended quality when the ground truth is generated by a combination of several commercially available enhancement methods. In the following, Equation (6) was used to calculate VGG loss.

(6) $$L_{VGG}=\frac{1}{WHC}\|F_{VGG}\left(\hat{I}\right)-F_{VGG}{\left(I\right)\|}^{2}$$

where W, H, and C represent a picture’s width, height, and depth, respectively. Specifically, the mean squared error was used to evaluate the gap between these elements.

In this case, SSIM loss outperforms L1 and L2 as loss functions because it simultaneously measures the brightness, contrast, and structural diversity. The following is an equation (Equation (7)) of the SSIM loss function:

(7) $$L_{SSIM}=1-SSIM\left(\hat{I},\;I\right)$$

These three loss functions are combined to form Equation (8) that is as follows:

(8) $$L=L_{SSIM}+\lambda_{vl}\cdot L_{VGG}+\lambda_{sl}\cdot L_{SMOOTH}$$

**Figure 5:**
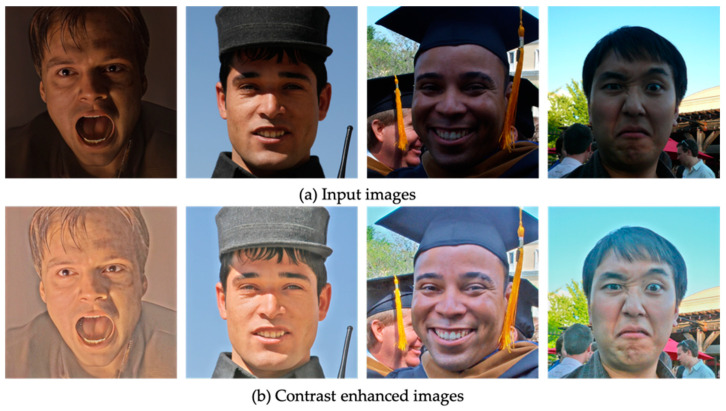
Example images of low-light image enhancement algorithm using AffectNet dataset.

Two popular image datasets [72,73] were used to train the low-light image improvement model. During individual −1E and −2E branch training, CC was first set to zero and then gradually increased to 0.1 in the joint training phase. DD, in contrast, was held constant at 0.1 throughout the process. Each dataset was split into a training set and an evaluation set. The results of the low-light image enhancement model are illustrated in Figure 5. The image enhancement algorithm’s dark lighting results were fed into a model for recognizing facial expressions.

#### 3.2.2. Recognizing Emotions from Masked Facial Images

**Figure 6:**
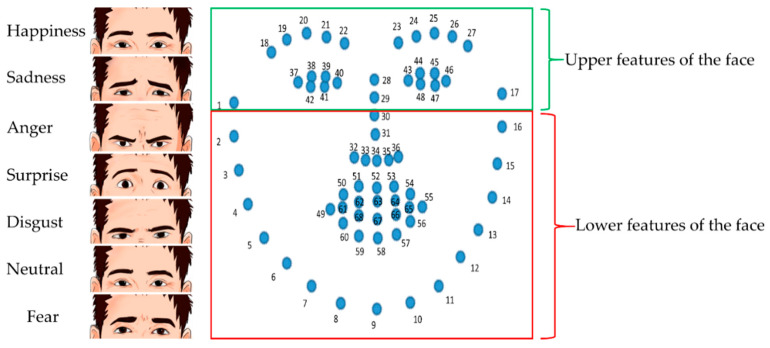
The standard 68 facial landmarks and seven basic emotions. (Upper and lower facial features.)

Most studies explore seven types of facial emotions: Happiness, Sadness, Anger, Surprise, Disgust, Neutral, and Fear, as displayed in Figure 6. Eye and eyebrow shape and pattern may help differentiate “Surprise” from the other emotions while the bottom features of the face (cheeks, nose, and lips) are absent. It is hard to describe the difference between “Anger” and “Disgust”. These two expressions can be confused because the top features of the face are almost the same as the bottom features; these two emotions may be correctly identified using a large wild dataset. The lower parts of the face are significant in conveying happiness, fear, and sadness, making it difficult to tell these correct emotions without them.

**Figure 7:**
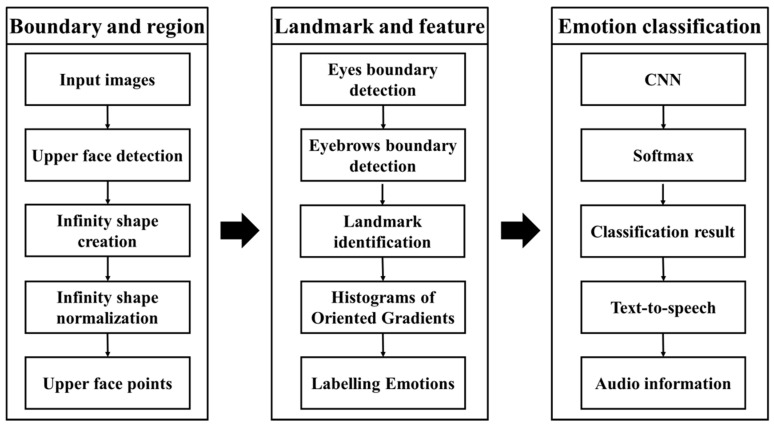
The general process of the proposed facial emotion recognition approach.

Necessary details can be gathered from the upper features of the face when the bottom features of the face are obscured. Since the top features of the face are affected by information spreading from the bottom area of the face, we concentrated on the eye and eyebrow regions. For creating top face points and feature extractions, we followed the work of Khoeun et al. [45]. An illustration of the facial emotion recognition approach from the bottom part of the face-masked images is shown in Figure 7.

#### 3.2.3. Generating and Detecting Synthetic Masked Face

**Figure 8:**
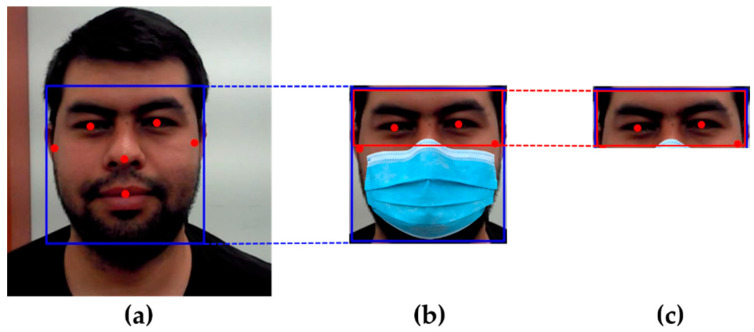
Generating and detecting synthetic masked face; (**a**) face regions detected using MediaPipe face detection; (**b**) the face is covered with synthetic facial mask using MaskTheFace; (**c**) detected upper features of the face.

This stage involved carrying the original facial emotion images from the AffectNet [24] dataset and utilizing it to build masked face images with artificial masks. To isolate the human face from an image’s background, we used MediaPipe face detection [74]. The face region size in each image was extracted correspondingly. Subsequently, the bottom part of the identified face, such as the cheeks, nose, and lips, were masked using the MaskTheFace approach [25], as illustrated in Figure 8. This created face images with artificial masks that were as realistic as possible. In the end, the top feature of the masked face picture was applied for further processing. Across the AffectNet dataset, the typical picture size was 425 pixels in both dimensions.

#### 3.2.4. Infinity Shape Creation

We set out to solve the problem of obstructed lower facial features by creating a fast facial landmark detector. We found that during emotional expression, the uncovered areas of the face (the eyes and eyebrows) became wider in contrast to the obscured areas (the lips, cheeks, and nose) [45]. To further aid in emotion classification, we aimed to implement a landmark identifier and select the face details vectors that indicate the crucial connections among those areas. We implemented this step to guarantee that the produced points covered the eyebrows and eyes. Instead of using the complete pixel region for training and classifying distinct emotions, it is necessary to identify the significant properties that occur between the lines linking neighborhood locations. Consequently, the computational complexity is drastically decreased.

#### 3.2.5. Normalizing Infinity Shape

The initial collection of points used to produce the infinity shaper is a different scale than the upper part of the face image. Before placing the initial infinity shape in its final place, it must be scaled to the correct dimensions so that it adequately covers the whole upper part of the face. The original x or y coordinate value is transformed into a different range or size according to each upper part of the face, as indicated in Equations (9) and (10), which allow us to determine the new coordinates of every position. Here, the upper part of the face width or height is the new range (max_new and min_new). As a result, the x and y coordinates were normalized, where the infinity shape’s size was comparable to that of the upper part of the face. Moreover, this is one of the many adaptable features of the method. Each area of the upper part of the face is measured, and then the original position set is normalized based on the average of these measurements.

(9) $$x_{normalized}=\frac{x_{original}-min_{x_{original}}}{max_{x_{original}}-min_{x_{original}}}\left(max_{x\_new}-min_{x\_new}\right)+min_{x\_new}\;y_{normalized}=\frac{y_{original}-min_{y_{original}}}{max_{y_{original}}-min_{y_{original}}}\left(min_{y\_new}-min_{y\_new}\right)+min_{y\_new}$$

#### 3.2.6. Landmark Detection

**Figure 9:**
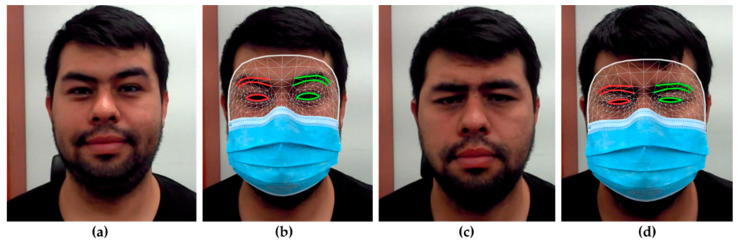
Results of facial landmark detection. (**a**) Happiness emotion; (**b**) landmark detection on masked face emotion; (**c**) sadness emotion; (**d**) landmark detection on masked face.

In order to identify masked and unmasked faces, we created a landmark detection technique. At this point, we used a model that detects landmarks on both masked and unmasked faces. Following this, MediaPipe was used to construct a deep learning framework. MediaPipe is a toolkit for developing machine learning pipelines for processing video streams. The MediaPipe face-mesh model [26] we used in our study calculates 468 3D facial landmarks, as displayed in Figure 9.

Using transfer learning, researchers at Facebook created a machine-learning model called MediaPipe face mesh [26]. MediaPipe face mesh was developed with the goal of recognizing the three-dimensional topology of a user’s face. The network architecture of the face mesh was developed on top of the blaze face [75] concept. The blaze face model’s primary function is detecting and estimating faces inside a given picture or video frame using bounding boxes. Face mesh estimates 3D coordinates after blaze face bounding boxes have been used to encircle the face. Two versions of deep neural networks run in real-time and make up the pipeline. The first part is a face detector that processes the entire image and calculates where faces are located. This second part is the 3D face landmark model that uses these points to construct a regression-based approximation of the 3D face surface.

To extract the eye-related features, it is necessary first to find the region of interest of both the right and left eyes. Every localization of a landmark in a facial muscle has a powerful connection to other landmarks in the same muscle or neighboring muscles. In this study, we observed that exterior landmarks had a detrimental impact on the accuracy of face emotion identification. Therefore, we employed the MediaPipe face mesh model to identify landmarks for the upper features of the face, such as eyes and eyebrows, where landmarks were input characteristics, intending to improve the model’s performance. In the following (Equation (10)), the facial landmarks are calculated:

(10) $$FL=\left\{x_{l,f},y_{l,f}|1\leq l\leq L,1\leq f\leq F\right\}$$

where *FL* describes a set of facial landmarks and $x_{l,f},y_{l,f}$ are the locations of each facial landmarks. *L* and *F* indicate the number of facial landmarks and image frames, respectively.

#### 3.2.7. Feature Extraction

In this step, we evaluated and selected important features of the upper features of the face. Figure 9 demonstrates that the majority of the observed upper features of facial landmarks are within the confines of the eye and eyebrow regions. The link between the identified landmarks and the landmarks’ individual coordinates are important elements for the categorization procedure. There are still some outliers, so the identified locations are treated as potential landmarks. In addition to completing the feature extraction procedure, this stage aims to eliminate the unimportant elements. To do this, we applied the histograms of the oriented gradients (HOG) from Equations (11)–(14) to all of the potential landmarks on the upper features of the face to obtain the orientation of the landmarks and their relative magnitudes.

(11) $$G_{x}\left(y,x\right)=I\left(y,x+1\right)-I\left(y,x-1\right)$$

(12) $$G_{y}\left(y,x\right)=I\left(y+1,x\right)-I\left(y-1,x\right)$$

(13) $$G\left(y,x\right)=\sqrt{G_{x}{\left(y,x\right)}^{2}+G_{y}{\left(y,x\right)}^{2}}$$

(14) $$\theta \left(y,x\right)=arctan\left[\frac{G_{y}\left(y,x\right)}{G_{x}\left(y,x\right)}\right]$$

Each landmark’s coordinates consist of its location (*y* and *x*) and 72 values representing the directional strength and blob size (20 by 20 pixels) all over the facial landmark. As a result, each landmark can include the 74-feature vector. In this way, the meaningful data collected from each landmark will include details on the connections between the many points of interest in a small area. Among qualitative identifiers, the HOG’s goal is to normalize attributes of every item, such that the identical items always yield the same feature identifier regardless of context. The HOG uses significant values in the local gradient vectors in duration. It uses the standardization of local histograms, the image’s gradient, and a set of histogram orientations for several places. It is also essential to restrict the local histograms to the region of the block. The basic assumption is that the distribution of border direction (local intensity gradients) determines local object appearance and shape, even if there are no hints to explicate the border placements or the corresponding gradient. The HOG characteristics that have been discovered for each landmark are combined into one vector. When a landmark’s *x* and *y* locations are combined with the HOG feature vector, the resulting information is assumed to be representational of that landmark. Before moving on to the classification stage, each image’s landmark information with a distinct emotion is categorized.

#### 3.2.8. Emotion Classification

**Figure 10:**
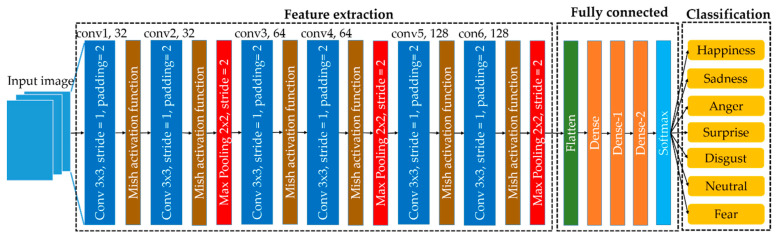
The network architecture of the emotion classification model.

In order to classify emotions, information from all landmarks is gathered collectively and sent into the training stage. We used CNNs, the Mish activation function, and the Softmax function for classification, as seen in Figure 10. Due to the many locations and characteristics in each facial image, all of the data are taken into account within a single training frame. The network architecture of a CNN design optimized for recognizing different facial emotions is shown in Figure 10. CNNs rely on their central convolution layer to best represent local connections and value exchange qualities. Each convolution layer was generated using the input picture and many trainable convolution filter methods, including the batch normalization approach, Mish activation function, and max pooling parameters, all of which were also used in the feature extraction of the emotion recognition model.

The batch normalization method was employed to decrease the time spent on learning by normalizing the inputs to a layer and maintaining the learning process of the algorithms. Without the activation function, the CNN model has the characteristics of a simple linear regression model. Therefore, the Mish activation function was employed in the network to learn complicated patterns in the image data. Furthermore, to reach the substantially more nuanced view region afforded by deep description, researchers need an activation function to build nonlinear connections between inputs and outputs. Even though leaky ReLU is widely used in deep learning, Mish often outperforms it. The use of Mish is a significant development that has the potential to enhance categorization precision. In the following (Equation (15)), the Mish activation function is described:

(15) $$y_{mish}=x\;\tan h\left(\ln\left(1+e^{x}\right)\right),$$

The leaky ReLU activation function is calculated as follows:

(16) $$y_{leaky\;relu}=\left\{\begin{array}{ll} x, & if\;x\geq 0\\ \lambda x, & if\;x<0 \end{array}\right.$$

The maximum value in each area of the facial feature map was then determined using a max-pooling process. We reduced the feature map’s dimensionality during the pooling process by moving a two-dimensional filter over each feature map. To avoid the need for exact feature positioning, the pooling layer summed the features contained in an area of the feature map created by the convolution layer. By reducing the number of dimensions, the model becomes less sensitive to shifts in the locations of the elements in the input data. The final layer of the proposed CNN model utilized a Softmax classifier, which can predict seven emotional states, as illustrated in Figure 10.

## 4. Experimental Results and Analysis

This part details the methodology used to test the facial emotion recognition model and the outcomes obtained. Facial emotion recognition datasets were utilized for both training and testing purposes. Significant features for the training stage include a learning rate of 0.001, a batch size of 32 pixels, and a subdivision of 8. Investigating the classifier’s performance is essential for developing a system that can consistently and correctly categorize facial emotions from masked facial images. This research examines and analyzes the performance of the proposed and other nine facial emotion recognition models for the purpose of accuracy comparison. In evaluation, it was determined that the proposed model accurately recognizes more facial emotions than other models. The results indicate that the proposed model successfully recognizes emotion in the wild. Both qualitative and quantitative analyses were employed to determine the experimental results.

To conduct this study, we utilized the AffectNet [24] database. The AffectNet database is one of the biggest and widely used natural facial expression emotion datasets for wild facial emotion recognition models and consists of 420,299 manually annotated facial images. Following the lead of the vast majority of previous studies [52,75,76,77,78,79,80], we chose to train our models using seven emotions and leave contempt out of the scope (more than 281,000 training images). We conducted our analyses using the validation set, which consisted of 500 images for each emotion and 3500 images in total. We randomly selected pictures for the test set from the remaining dataset. It is worth noting that the deep learning model is trained from original images by scaling them into a size of 512 by 512 without cropping the images of faces, so much of the surrounding elements, hair, hats, or hands are present. We mainly did this to ensure that the images are wild and similar to real-life scenarios. During the training process, we employed the stochastic gradient descent optimizer.

Images from this widely used database were chosen due to their wide variety of subjects close to real-world illumination, head orientation, interferences, gender, age range, and nationality. The AffectNet database, for example, includes people aged 2 to 65 from a range of countries. Approximately 60% of the photos are of women, giving a rich diversity of perspectives and a challenging task. It allowed us to investigate real-world photographs of people’s faces which are rich with anomalies. Online web sources were searched for the images. Two annotators each marked a set of images with simple and complex keywords. In this study, we only utilized the standard emotion images for the seven fundamental emotions: Happiness, Sadness, Anger, Surprise, Disgust, Neutral, and Fear. All of the photos were 256 pixels per side after being aligned. Facial hair, age, gender, nationality, head position, eyeglasses and other forms of occlusion, and the effect of illumination and other post-processing techniques, including different image filters, all contribute to a great deal of visual diversity throughout the available pictures. The participants in this data set were Asian, European, African American, and Caucasian. The dataset comprised individuals aged 2–5, 6–18, 19–35, 36–60, and 60+.

Instead of relying on an embedded approach, which may not be the best choice for boosting the power storage viability of smart glasses and securing the system’s real-time execution, it is preferable to use a high-performance AI server [22]. Whether or not the designed smart glasses system is implemented depends on how well the AI server executes. This is because smart glasses designs often use deep learning models, which need significant processing power on the AI server. All experiments were run on an AI server, and the specifications of the server are summarized in Table 3.

A client component, including smart glasses and a smartphone, sent acquired photos to the AI server. After that, computer vision and deep learning models were used to process the input photos. The outcomes were transmitted to the client component via Wi-Fi/Internet so the user may listen to the output sounds over headphones/speakers. The following are the qualitative and quantitative experimental outcomes of the deep learning models implemented on the AI server.

### 4.1. Qualitative Evaluation

**Figure 11:**
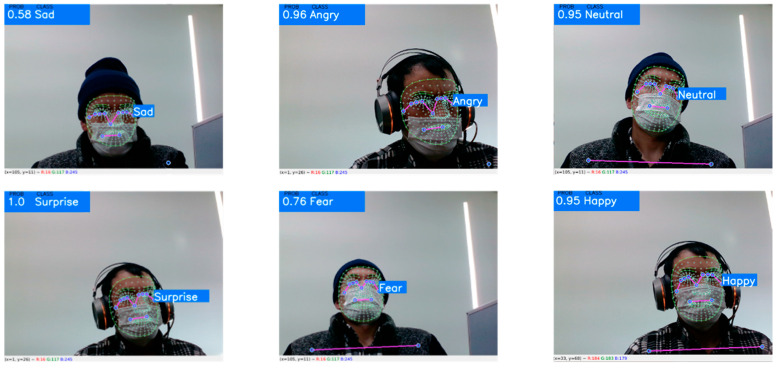
Classification results of the proposed facial emotion recognition.

Initially, we assessed the proposed facial emotion recognition model qualitatively. We used a computer’s web camera for a live video stream for this experiment. Figure 11 shows the classification results of the facial emotion recognition model from a masked face in a real-time environment. In the upper left corner of the image, the percentage of classification along with the recognized facial emotion is shown, and on the right side of the mask, the class of the facial emotion is illustrated.

This demonstrates that the proposed facial emotion recognition model successfully labelled seven facial expressions. As a programmable module, it may be included in smart glasses [31] to help BVI people to perceive people’s emotions in social communication.

### 4.2. Quantitative Evaluation Using AffectNet Dataset

This work evaluated the classification approaches for facial emotion recognition using quantitative evaluation procedures. Quantitative experiments were carried out, and the results were analyzed using widespread object detection and classification evaluation metrics such as accuracy, precision, sensitivity, recall, specificity, and F-score, as mentioned in previous works [81,82,83,84]. Precision measures how well a classifier can separate relevant data from irrelevant data or the proportion of correct identifications. The percentage of true positives measures a model’s precision in spotting relevant circumstances it identifies within all ground truths. The suggested method was compared to its findings using ground-truth images at the pixel level, and precision, recall, and F-score metrics were calculated. Accuracy (*AC*), precision (*PR*), sensitivity (*SE*), specificity (*SP*), recall (*RE*), and F-score (*FS*) metrics for the facial emotion recognition systems were determined using the following equations:

(17) $$AC_{C_{ij}}=\frac{TP_{C_{ij}}+TN_{C_{ij}}}{TP_{C_{ij}}+TN_{C_{ij}}+FP_{C_{ij}}+FN_{C_{ij}}},$$

(18) $$PR_{C_{ij}}=\frac{TP_{C_{ij}}}{TP_{C_{ij}}+FP_{C_{ij}}},$$

(19) $$SE_{C_{ij}}=\frac{TP_{C_{ij}}}{TP_{C_{ij}}+FN_{C_{ij}}},$$

(20) $$SP_{C_{ij}}=\frac{TN_{C_{ij}}}{TN_{C_{ij}}+FP_{C_{ij}}},$$

(21) $$RE_{C_{ij}}=\frac{TP_{C_{ij}}}{TP_{C_{ij}}+FN_{C_{ij}}},$$

where *TP* = number of true positive samples; *TN* = number of true negative samples; *FP* = number of false positive samples; *FN* = number of false negative samples; and *C* = number of categories. We also calculated the F-score value, which measures how well accuracy and recall are balanced. It is also named the F1 Score or the F Measure. The F-score was defined as follows, where a larger value indicates better performance:

(22) $$FS=\frac{2*RE*PR}{RE+PR}$$

Table 4 shows comparison results of the proposed and other models. In general, as indicated in Table 4, the proposed model obtained the highest result, with 69.3% accuracy for the images derived from the AffectNet dataset. Figure 9 also compares the precision, recall, F-score, and accuracy of the proposed model’s application to the AffectNet datasets.

The performance in Table 4 confirms that the proposed model ranks first on the AffectNet dataset, with an accuracy of 69.3%. This was followed by the models of Farzaneh et al. [52] and Shi et al. [53], which obtained an accuracy of 65.2% (a difference of 4.1% from the proposed model).

**Figure 12:**
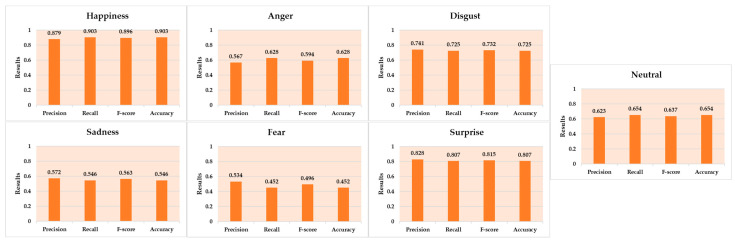
Evaluation results of the seven emotions for the proposed method on the AffectNet dataset.

According to our findings, identifying people’s emotional expressions when a mask covers the lower part of the face is more problematic than when both parts of the face are available. As expected, we discovered that the accuracy of expression detection on a masked face was lower across all seven emotions (Happiness, Sadness, Anger, Fear, Disgust, Neutral, and Surprise). Table 5 and Figure 12 illustrate the results of the suggested emotion recognition method across seven different emotion categories. Facial landmarks such as eyebrows and eyes move and change more in Happiness and Surprise; therefore, the results for these emotions are the most accurate, with 90.3% and 80.7% accuracy, respectively. However, Fear, Sadness, and Anger resulted in minor adjustments to the eyebrow and eye landmarks, obtaining of 45.2%, 54.6%, and 62.8% accuracy, respectively. Although the landmark features associated with the emotions of Fear and Disgust (the eyebrows and eyes) are identical, an accuracy of 72.5% was reached.

### 4.3. Evaluation Based on Confusion Matrix

**Figure 13:**
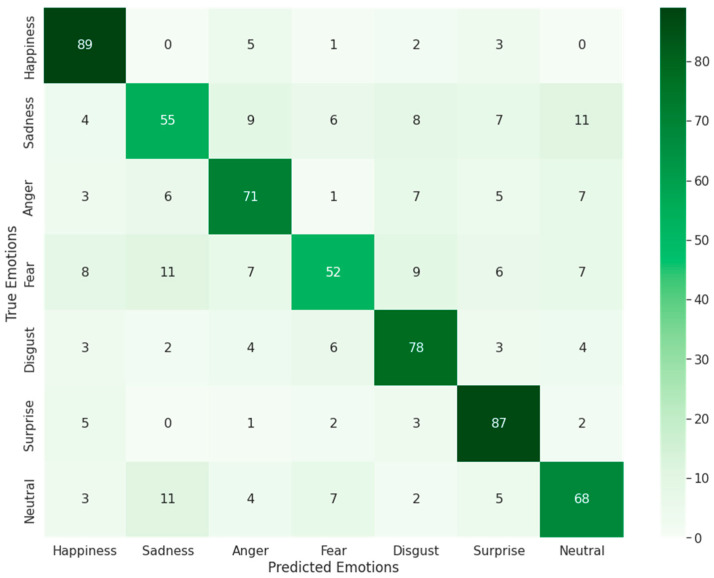
Performance of a confusion matrix for facial emotion recognition using the proposed model on the AffectNet dataset.

Furthermore, as seen in Figure 13, the suggested model was assessed by employing a confusion matrix for facial emotion recognition. For each of the seven categories, the authors chose 100 facial emotion photos randomly. Roughly 80% of randomly chosen photos depict a single subject on a simple background, while the remaining 20% depict wild scenes with complex backgrounds.

The assessment findings indicate that the proposed approach has an accuracy of 69.3%, and the average result of the confusion matrix is 71.4%.

## 5. Conclusions

In this study, we employed deep CNN models and facial landmark identification to recognize an individual’s emotional state from a face image where the bottom part of the face is covered with a mask. AffectNet, a dataset of images labeled with seven basic emotions, was used to train the suggested face emotion identification algorithm. During the studies, the suggested system’s qualitative and quantitative performances were compared to other widespread emotion recognizers based on facial expressions in the wild. The results of the experiments and evaluations showed that the suggested method was effective, with an accuracy of 69.3% and an average confusion matrix of 71.4% for the AffectNet dataset. Assistive technology for the visually impaired can greatly benefit from the suggested facial expression recognition method.

Despite the accuracy mentioned above, the work has some limitations in the various orientation scenarios. Facial landmark features were not correctly obtained due to an orientation issue. Furthermore, the proposed model also failed to recognize emotions when multiple faces were present in the same image at an equal distance from the camera.

The authors plan to further refine the classification model and picture datasets by investigating methods such as semi-supervised and self-supervised learning. As attention CNN relies on robust face identification and facial landmark localization modules, we will investigate how to produce attention parts in faces without landmarks. In addition, we plan to work on the hardware side of the smart glasses to create a device prototype that can help the visually impaired identify people, places, and things in their near surroundings.

## Figures and Tables

**Table 1:** The comparison of existing facial emotion recognition methods.

Models	Facial Features	Emotions	Datasets	Recognition in Dark
ExNet [48]	Upper and lower	7	FER-2013, CK+, RAF-DB	No
Shao et al. [49]	Upper and lower	7	CK+, FER-2013	No
Miao et al. [50]	Upper and lower	7	FER2013, CASME II, SAMM	No
Wang et al. [51]	Upper and lower	8	FERPlus, AffectNet, RAF-DB	No
Farzaneh et al. [52]	Upper and lower	7	RAF-DB, AffectNet	No
Shi et al. [53]	Upper and lower	8	RAF-DB, AffectNet	No
Li et al. [54]	Upper and lower	7	RAF-DB	No
Li et al. [55]	Upper and lower	7	RAF-DB, AffectNet	No
Khoeun et al. [45]	Upper	8	CK+, RAF-DB	No
Our previous work [56]	Upper	7	FER-2013	No
The proposed work	Upper	7	AffectNet	Yes

**Table 2:** A comparison of existing facial emotion recognition datasets.

Datasets	Total Size	Image Size	Emotion Categories	Number of Subjects	Condition
MultiPie [57]	750,000	3072 × 2048	6	337	Controlled
Aff-Wild [68]	10,000	Various	Valence and arousal	2000	Wild
Lusey et al. [58]	10,708	~	7	123	Controlled
Pantic et al. [60]	1500	720 × 576	6	19	Controlled
AM-FED [61]	168,359	224 × 224	14 action unit	242	Spontaneous
DISFA [62]	130,000	1024 × 768	12 action unit	27	Spontaneous
FER-2013 [65]	~35,887	48 × 48	7	~35,887	Wild
AFEW [69]	Videos	Various	7	130	Wild
EmotionNet [70]	1,000,000	Various	23	~10,000	Wild
FER-Wild [71]	24,000	Various	7	~24,000	Wild
AffectNet [24]	1,000,000	Various	8	~450,000	Wild

**Table 3:** The hardware and software specification of the AI server.

Components	Specifications	Descriptions
GPU	GPU 2-GeForce RTX 2080 Ti 11 GB	Two GPU are installed
CPU	Intel Core 9 Gen i7-9700k (4.90 GHz)	
RAM	DDR4 64 GB (DDR4 16 GB × 4)	Samsung DDR4 16 GB PC4-21300
Storage	SSD: 512 GB/HDD: TB (2 TB × 2)	
Motherboard	ASUS PRIME Z390-A STCOM	
OS	Ubuntu Desktop	Version: 18.0.4 LTS

**Table 4:** Comparison of facial emotion recognition model performance on the AffectNet dataset.

Models	Accuracy	Models	Accuracy
Wang et al. [51]	52.97%	Li et al. [86]	58.89%
Farzaneh et al. [52]	65.20%	Wang et al. [87]	57.40%
Shi et al. [53]	65.2%	Farzaneh et al. [88]	62.34%
Li et al. [55]	58.78%	Weng et al. [89]	64.9%
Li et al. [85]	55.33%	Proposed model	69.3%

**Table 5:** The results of evaluation metrics for the proposed method on the AffectNet dataset.

Facial Emotions	Precision	Sensitivity	Recall	Specificity	F-Score	Accuracy
Happiness	87.9%	89.4%	90.3%	90.8%	89.6%	89
Sadness	57.2%	58.7%	54.6%	55.2%	56.3%	54.6%
Anger	56.7%	57.3%	62.8%	63.5%	59.4%	62.8%
Fear	53.4%	54.2%	45.2%	45.7%	49.6%	45.2%
Disgust	74.1%	74.8%	72.5%	73%	73.2%	72.5%
Surprise	82.8%	83.5%	80.7%	81.4%	81.5%	80.7%
Neutral	62.3%	63.1%	65.4%	65.9%	63.7%	65.4%

## Data Availability

All open access datasets are cited with reference numbers.
